# Combinations of TLR Ligands: A Promising Approach in Cancer Immunotherapy

**DOI:** 10.1155/2013/271246

**Published:** 2013-11-25

**Authors:** Saskia Stier, Claudia Maletzki, Ulrike Klier, Michael Linnebacher

**Affiliations:** Department of General Surgery, Molecular Oncology and Immunotherapy, University of Rostock, Schillingallee 35, 18057 Rostock, Germany

## Abstract

Toll-like receptors (TLRs), a family of pattern recognition receptors recognizing molecules expressed by pathogens, are typically expressed by immune cells. However, several recent studies revealed functional TLR expression also on tumor cells. Their expression is a two-sided coin for tumor cells. Not only tumor-promoting effects of TLR ligands are described but also direct oncopathic and immunostimulatory effects. To clarify TLRs' role in colorectal cancer (CRC), we tested the impact of the TLR ligands LPS, Poly I:C, R848, and Taxol on primary human CRC cell lines (HROC40, HROC60, and HROC69) *in vitro* and *in vivo* (CT26). Taxol, not only a potent tumor-apoptosis-inducing, but also TLR4-activating chemotherapeutic compound, inhibited growth and viability of all cell lines, whereas the remaining TLR ligands had only marginal effects (R848 > LPS > Poly I:C). Combinations of the substances here did not improve the results, whereas antitumoral effects were dramatically boosted when human lymphocytes were added. Here, combining the TLR ligands often diminished antitumoral effects. *In vivo*, best tumor growth control was achieved by the combination of Taxol and R848. However, when combined with LPS, Taxol accelerated tumor growth. These data generally prove the potential of TLR ligands to control tumor growth and activate immune cells, but they also demonstrate the importance of choosing the right combinations.

## 1. Introduction

Since the last decades of cancer research, numerous approaches have been initiated aiming at activating cytotoxic immune reactions against tumors. Besides targeting the adaptive immune system, stimulators of the innate immune system gained much attention. In this context and resulting from their strong immune stimulatory capacity, ligands for Toll-like receptors (TLRs) were extensively studied. TLRs are a family of pattern recognition receptors. They have a key position in the first-line defense against pathogens by recognizing specific pathogen-associated molecular patterns, conserved structures expressed by pathogens. Furthermore, they bind to endogenous damage-associated molecular patterns. These molecules are released by stressed or dying cells [1]. Upon ligand binding, TLR-signaling leads to inflammation and antimicrobial responses, thus priming adaptive immune responses [2]. Besides components directly originating from bacteria or viruses, synthetic substances like Poly I:C (ligand for TLR3) or Resiquimod (R848, ligand for TLR7/8) were extensively studied either as single substance in experimental cancer models or as vaccine adjuvant in clinical trials [3–5].

TLRs are primarily expressed by cells belonging to the innate immune systems' arm, that is, dendritic cells (DCs) and monocytes. The observation that TLRs are functionally expressed in several types of tumors, however, hints towards another tumor-promoting role [6]. Recent evidence suggests that they act as double-edged sword in tumorigenesis.

Even more, several studies revealed adverse effects of TLRs on carcinogenesis. Kundu et al. described LPS-induced malignant transformation of benign prostate epithelia [7]. The group of Schmaußer found that TLRs on malignant gastric carcinoma cells enabled interaction with pathogens and subsequently enhanced cell proliferation [8]. Some additional studies substantiated a tumor growth and malignancy-promoting effect of TLRs overexpressed on tumor cells. These include employment of TLR4 signaling by breast and colorectal cancer cells [9, 10], as well as flagellin-induced activation of TLR5 on gastric cancer cells [11]. 

On the other hand, at least as many studies revealed antitumoral effects of TLR ligands by inducing tumor cell apoptosis/necrosis or activating immune cells. Direct oncopathic effects on different tumor entities have been described for Poly I:C (TLR3 agonist) and Imiquimod (TLR7 agonist) [12–14]. Hence, similar to what is known about the immune system as a whole, TLRs are capable of both inhibiting and promoting cancer.

Although the TLR expression patterns and their effects are well understood on immune cells, their functional relevance in tumorigenesis and resulting immunological changes remain to be fully elucidated. Further studies are needed to clarify their function in tumor biology and to evaluate their therapeutic potential which will finally help to establish effective treatment schedules. Therefore, we here tested TLR ligands for treatment of colorectal carcinomas (CRC) *in vitro* and *in vivo*. Experiments revealed strongest oncolytic effects in the presence of a functional immune system. Hence, these findings underscore the rationale for using TLR ligands in cancer immunotherapy—either alone or as combinations; preferably together with conventional chemotherapy.

## 2. Material and Methods

### 2.1. Tumor Cell Lines and TLR Ligand Treatment

The CRC cell lines HROC40, HROC60, and HROC69 (all three microsatellite stable) were established in our lab from patients subsequent to operation and for analyses passages 25–35 were used. Cells were maintained in full medium: DMEM/HamsF12 supplemented with 10% fetal calf serum, glutamine (2 mmol/L), and antibiotics (medium and supplements were purchased from PAA, Cölbe, Germany). The cells were seeded at the appropriate density for each cell line in both 96-well or 24-well plates and incubated 24 h prior to TLR ligand treatment. For all *in vitro* experiments, the following TLR ligands and their combinations were used in the concentrations 0.01, 0.1, 1, and 10 *μ*M: Taxol, R848 (Enzo Life Sciences, Lörrach, Germany), LPS (Sigma-Aldrich, Hamburg, Germany), and Poly I:C (InvivoGen, San Diego, CA, USA). All substances were applied once. Antitumoral effects were examined after 24, 48, and 72 h of incubation.

### 2.2. RNA Isolation, cDNA Synthesis, and Quantitative Real-Time PCR

Total RNA from tumor cells was isolated with TRIzol reagent according to the manufacturer's instructions. RNA was reverse-transcribed into cDNA from 2 *μ*g RNA using the High Capacity cDNA Reverse Transcription Kit (Applied Biosystems, Foster City, CA, USA). Target cDNA levels of human cell lines were analyzed by quantitative real-time PCR using TaqMan Universal PCR Master Mix and predesigned TaqMan gene expression assays, Hs00152933_m1 (TLR3), Hs00152939_m1 (TLR4), Hs00152971_m1 (TLR7), Hs00152972_m1 (TLR8), Hs00152973_m1 (TLR9), and Hs99999905_m1 (GAPDH, housekeeping gene control) in an ABI Prism 7000 sequence detection system (Applied Biosystems). PCR conditions were as follows: 95°C for 10 min, 40 cycles of 15 s at 95°C, and 1 min at 60°C. TLR expression by the murine CRC cell line CT26 was analyzed using SibirRoxHot Master Mix (Bioron, Ludwigshafen, Germany). The mRNA levels of target genes were normalized to GAPDH. Primer pairs used in real-time PCR were the following: TLR3 5′-GGTCCCCAGCCTTCAAAGAC-3′ and 5′-ACGAAGAGGGCGGAAAGGT-3′, TLR4 5′-ACCTGGCTGGTTTACACGTC-3′ and 5′-CTGCCAGAGACATTGCAGAA-3′, TLR7 5′-CCACAGGCTCACCCATACTTC-3′ and 5′-GGGATGTCCTAGGTGGTGACA-3′, GAPDH 5′-CATGGCCTTCCGTGTTCCTA-3′ and 5′-CCTGCTTCACCACCTTCTTGAT-3′. Reactions were performed in triplicate wells. The general expression level of each sample was considered by calculating ΔCT (ΔCt = Ct_target_ − Ct_GAPDH_). Expression patterns were classified as strong, moderate, low, or absent in comparison to normal immune cells (i.e., human dendritic cells and murine macrophages). These cells served as standard and quality control in each qRT-PCR. 

### 2.3. MTS and Flow Cytometric Cell Viability Analysis

Experiments were performed in 96-well plates in triplicate and replicated at least three times. MTS (Promega, Mannheim, Germany) was mixed with PMS (Sigma-Aldrich) and 20 *μ*L of this mix was added to each well. After incubation of cells at 37°C for at least 1 h, the absorbance was measured at 492 nm on a LP400 ELISA reader (Anthos Mikrosysteme, Krefeld, Germany). Direct TLR ligand effects on tumor cell viability were additionally characterized by flow cytometry. Cells were treated as described above. All cells (adherent plus cells in supernatant) were harvested and stained with 2 *μ*M Calcein-AM (Sigma Aldrich). Fluorescent microsphere beads (1.4 × 10^5^ beads/mL, Polysciences, Germany) were added to the samples in a final volume of 200 *μ*L. A gate was set in the FSC/SSC on the beads, and all living cells (Calcein-AM positive) per 5.000 beads were counted. Experiments were performed in 24-well plates in duplicates and replicated at least four times. Percentages of proliferating/viable and total number of cells were calculated compared to untreated control cells, that is, without TLR ligand application. Samples were analyzed on a FACSCalibur Cytometer (BD Pharmingen). Data analysis was performed using CellQuest software (BD Pharmingen).

### 2.4. Lymphocyte Preparation and Coculture Experiments

Peripheral blood lymphocytes (PBL) were obtained from healthy volunteers following Ficoll density-gradient centrifugation. The cytotoxicity mediated by TLR ligand-stimulated immune cells on CRC cell lines was examined by direct coculture experiments. Tumor cells were seeded in duplicate into 24-well plates (1 × 10^4^/well) and incubated overnight. Medium was removed and fresh medium containing PBLs (1 × 10^6^ PBL/well, ratio 1 : 100) with or without TLR ligand was added. Following a 48 h or 72 h incubation period, PBLs were removed and tumor cells were harvested by trypsinization. Prior to FACS analysis, fluorescent microsphere beads (1.4 × 10^5^ beads/mL, Polysciences, Germany) were added to the samples in a final volume of 200 *μ*L. One gate was placed on tumor cells in the FSC/SSC to exclude lymphocytes. A second gate was set in the FSC/SSC on the beads, and all living tumor cells per 5.000 beads were counted. A representative plot is given in Figure 4(c). Data are given as X-fold number of tumor cells compared to untreated controls with PBLs. Another control consisted of tumor cells without PBL addition. 

### 2.5. Flow Cytometric Phenotyping of PBL

For phenotypic analysis of human PBLs, cells were harvested after TLR ligand stimulation as described before (72 h). Controls were incubated in complete medium without any TLR ligand. Cells were washed and stained with directly FITC-, PE-, or APC-labeled mAbs against CD3, CD4, CD8, CD16/56, CD25, CD62L, CD69, and CD71 (each 1 *μ*g, ImmunoTools, Friesoythe, Germany) for 30 min at 4°C. Then, cells were washed twice and resuspended in 200 *μ*L PBS. Negative controls were stained with the appropriate isotypes. Cells were analyzed by flow cytometry as described above. For each sample, 20.000 events were measured. To overcome interindividual differences between single donors, data of untreated control PBLs were set as 1 and data of TLR ligand stimulated cells were given as X-fold increase. 

### 2.6. *In Vivo* Tumor Models and Treatment Regimen

Experiments were performed on female 8–10-week-old Balb/c mice weighing 18–20 g. Mice were bred in the university's animal facility and maintained under specified pathogen-free conditions. All animals were fed standard laboratory chow and given free access to water. Experiments were performed in accordance with the German legislation on protection of animals and the Guide for the Care and Use of Laboratory Animals (Institute of Laboratory Animal Resources, National Research Council; NIH Guide, vol. 25, no. 28, 1996). Tumor challenge was performed by subcutaneous (s.c.) injection of 5 × 10^6^ CT26 cells into the right hind leg. Tumor growth was routinely controlled at least twice a week and tumor volume was estimated according to the formula: *V* = width^²^∗length∗0.52. After tumor establishment, mice were divided into experimental groups (*n* = 7 per group) each treated with one of the following substances/combinations: Taxol (20 mg/kg bw), R848 (60 mg/kg bw), LPS (2 mg/kg bw), Irinotecan (20 mg/kg bw), Taxol + R848, Taxol + LPS, and R848 + LPS. Treatment was performed two times a week for a total of three weeks. As control, tumor-carrying mice received equivalent volumes of PBS (saline, *n* = 7). Tumor-carrying mice (treatment, control) were sacrificed at day 21 or when they became moribund before the tumor volume reached 2.000 mm^3^. Blood samples were taken on day 10 of therapy. At the end of each experiment, blood samples, tumor material, spleen, and mesenteric lymph nodes were removed from all animals for further analysis.

### 2.7. Flow Cytometry of Blood and Spleen Cells

Flow cytometry was performed with leukocytes from peripheral blood during and after therapy using the following fluorescein-isothiocyanate- (FITC-) and phycoerythrin- (PE-) conjugated rat anti-mouse monoclonal antibodies (mAbs): CD3*ε* FITC, CD62L PE (1 *μ*g, ImmunoTools), CD11b FITC, CD11c FITC, CD19 FITC, CD4 PE, CD8 PE, Gr1 PE (1 *μ*g, Miltenyi Biotec, Bergisch-Gladbach, Germany), and CD166 PE (1 *μ*g eBiosciences, Frankfurt, Germany) followed by lysis of erythrocytes (FACS Lysing Solution, BD Pharmingen). Negative controls consisted of lymphocytes stained with the appropriate isotypes (BD Pharmingen). Samples were analyzed as described above.

### 2.8. Statistical Analysis

All values are expressed as mean ± SE for *in vitro* data and mean ± SEM for tumor growth data. After proving the assumption of normality, differences between controls and experimental samples were determined by using the unpaired Student's *t*-test. If normality failed, the nonparametric Mann-Whitney *U*-Test was applied. The tests were performed by using Sigma-Stat 3.0 (Jandel Corporation, San Rafael, CA, USA). The criterion for significance was set to *P* < 0.05.

## 3. Results

### 3.1. TLR Expression on CRC Cell Lines

As a starting point for this study, the expression of TLRs was analyzed by qPCR on a set of ultra-low-passage CRC cell lines established in our lab. According to the TLR ligands chosen for the subsequent functional analyses, TLR3 (Poly I:C), TLR4 (LPS, Taxol), TLR7, and TLR8 (both R848) were examined (Table 1). TLR8 was not expressed at all, TLR7 was expressed at low levels by all cell lines; TLR4 showed moderate expression in HROC40, HROC60, and HROC69 cells compared to expression patterns of DCs. Similarly, TLR3 expression varied between cells. 

### 3.2. Direct Effects of TLR Ligands on CRC Cells

To evaluate direct effects of TLR ligands R848, LPS, Poly I:C, and Taxol on CRC cells, the three primary tumor cell lines HROC40, HROC60, and HROC69 were treated with increasing concentrations, ranging from 0.01 *μ*M to 10 *μ*M. Readout was performed after 24, 48, and 72 hours using a standard MTS assay. In each experiment and at every given time point, untreated cells served as controls. 

**Figure 1:**
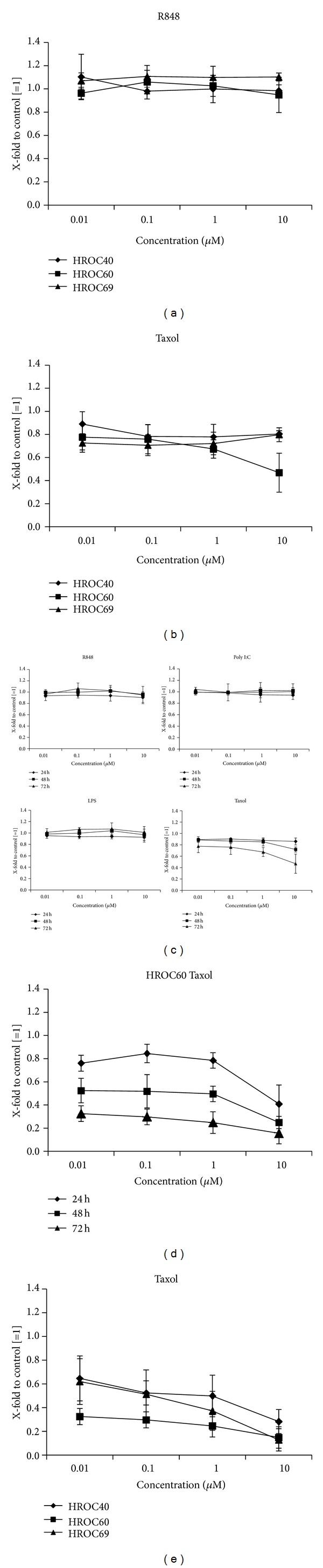
Direct cytotoxicity of TLR ligands towards CRC cell lines. Tumor cells were treated with increasing concentrations of the TLR ligands (a) R848 and (b) Taxol for 72 h. Cell viability was assessed by standard MTS assay. Results for the HROC60 cell line reached statistical significance (*P* < 0.05 versus control). (c) HROC60 cells were treated with TLR ligands for 24, 48, and 72 h with different concentrations (0.01–10 *μ*M). (d) HROC60 cells were treated with Taxol for 24, 48, and 72 h with different concentrations (0.01–10 *μ*M). Antitumoral effects were determined by a flow cytometric assay. Given results reached significance (*P* < 0.05 versus control). (e) The effect of increasing Taxol concentrations on CRC cell lines was assessed after 72 h incubation by flow cytometry. Results for HROC60 cells reached statistical significance at all concentrations; significant growth inhibition of HROC40 and HROC69 cells was obtained at 10 *μ*M and 1 *μ*M (*P* < 0.05 versus control). Untreated cells without TLR ligand were set as 1 and all data are given as X-fold increase. Experiments were performed in duplicate and repeated at least three times. Values are given as mean ± SD; *P* < 0.05 versus control; *t*-test.

The ligands for TLR7/8 (R848), TLR4 (LPS), and TLR3 (Poly I:C) exerted no significant antiproliferative effects (data not shown and exemplary results for R848 after 72 hours in Figure 1(a)). Taxol was the only cell growth inhibiting drug (Figure 1(b)); however, HROC40 and HROC69 displayed nearly complete resistance but in the highest dose (10 *μ*M), Taxol inhibited growth of HROC60 cells up to >50%. The metabolic activity was determined by a MTS assay and generally tended to decrease in a time- and dose-dependent manner (Figure 1(c)).

To prove these data, number and viability of CRC cells were analyzed after TLR ligand treatment by a flow cytometric assay. In principle, this test confirmed the MTS data in that Taxol was the only TLR ligand tested with direct antitumoral potential. Again, a clear time and dose dependency was observed in comparison to untreated control cells (Figure 1(d)). Antitumoral effects were generally more pronounced when compared to the results of the MTS assays. And here, Taxol exerted effects not only towards HROC60, but also against all three cell lines tested (Figure 1(e)). 

**Figure 2:**
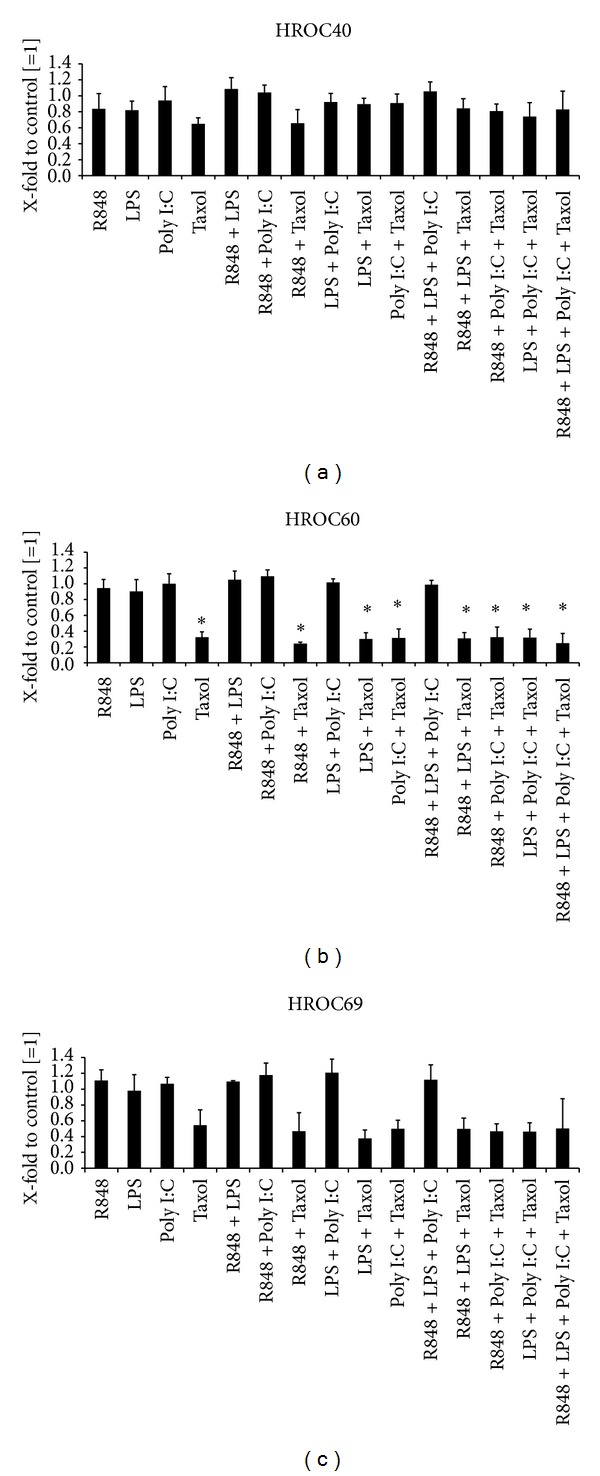
Direct cytotoxicity of TLR ligand combinations towards CRC cell lines. (a) HROC40, (b) HROC60, and (c) HROC69 cells were treated with all possible combinations between R848, LPS, Poly I:C, and Taxol for 72 h in a concentration of 0.01 *μ*M. Antitumoral effects were determined by flow cytometry. Results after treatment with single TLR ligands (0.01 *μ*M) are additionally shown. Untreated cells without TLR ligand were set as 1 and all data are given as X-fold increase. Experiments were performed in duplicate and repeated at least three times. Values are given as mean + SD; *P* < 0.05 versus control; *t*-test.

To analyze if any synergism of the TLR ligands on direct antitumoral effects can be observed, all possible combinations of the substances were tested in the lowest concentration (0.01 *μ*M). Readout was again performed by flow cytometry measuring the proportion of living cells in comparison to untreated controls. The antitumoral effect of Taxol towards HROC69 and HROC60 was slightly increased by any additional substance (Figures 2(a) and 2(b)). However, no increase could be observed for the cell line HROC40 (Figure 2(c)). Incubation with three or four substances showed no further enhancement of this effect (Figure 2). Similar to the results of the single agents, none of the combinations without Taxol exerted any antitumoral effect on the tumor cell lines (Figure 2).

### 3.3. Immune Stimulation by TLR Ligands

**Figure 3:**
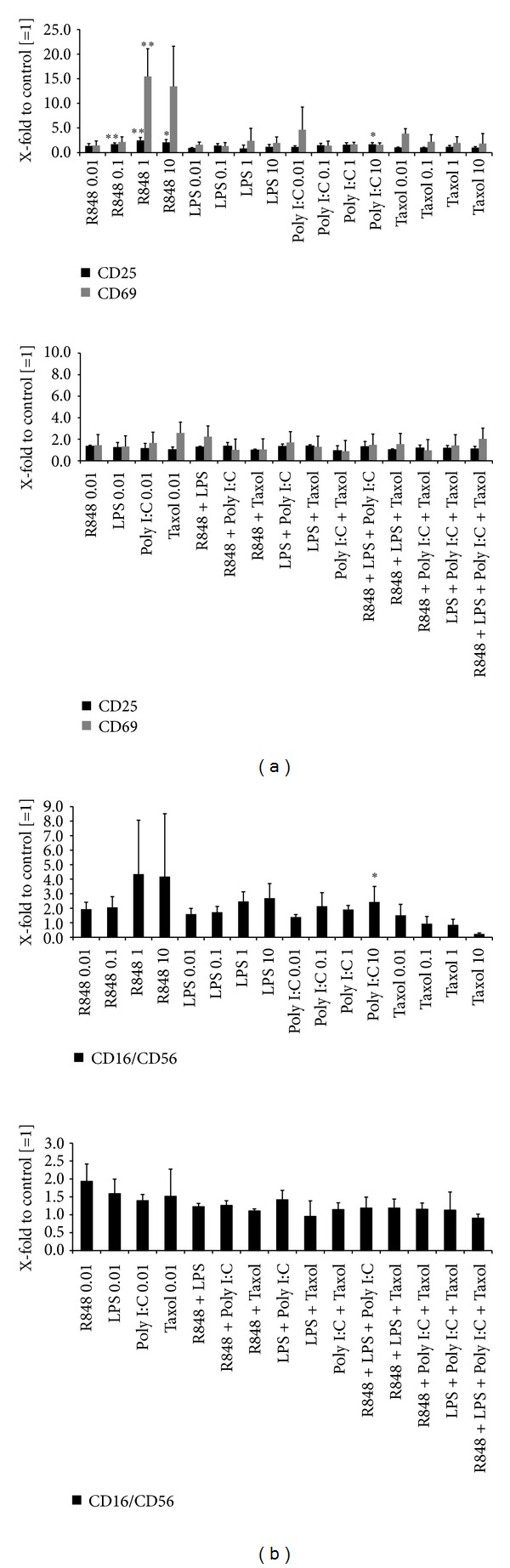
Flow cytometric phenotyping of TLR ligand stimulated PBLs. PBLs were either incubated with single TLR ligands (0.01 *μ*M; (a) and (b) upper panel) or in the presence of TLR ligand combinations (0.01 *μ*M; (a) and (b) lower panel) for 72 h. (a) Activation of PBLs following TLR stimulation as given by positive staining for CD25 and CD69. (b) CD16^+^CD56^+^ NK cells were the main responding cell population. Data of untreated control PBLs were set as 1 and all data were given as X-fold increase. Values are presented as mean + SD; *P* < 0.05 versus control; *t*-test.

TLR ligands exert direct immune stimulatory effects. To further elucidate their impact on PBLs in our setup, we performed a series of *in vitro* experiments. PBLs were either stimulated with single substances (all concentrations) or their combinations (each 0.01 *μ*M). As expected, TLR ligands directly stimulated immune cells. In detail, most pronounced effects were observed for R848. This substance activated immune cells in a dose-dependent manner (Figure 3(a), upper panel). Numbers of CD25^+^ and CD69^+^ activated cells increased upon TLR stimulation (Figure 3(a)). Likewise, proportions of CD16^+^CD56^+^ were elevated (Figure 3(b)). Hence, NK cells were identified as the main responding cell population. Poly I:C and Taxol exerted weaker though still stimulating effects, however, only at low concentrations. By contrast, LPS-mediated influences on PBLs could largely be neglected. When analyzing TLR ligand combinations, no further boost of immune stimulation was observed. 

### 3.4. Enhancement of TLR Ligand Mediated *In Vitro* Effects by Lymphocytes

The above results demonstrated no effects of the TLR ligands R848, LPS, and Poly I:C but a strong influence of Taxol on CRC cells. Since the main antitumoral effects of TLR ligands are likely to base on immune stimulation, we next analyzed the effects of TLR-stimulated immune cells on CRC cell lines. The latter were coincubated with PBL (ratio 100 : 1, PBL to tumor cell) from five healthy volunteers in the presence of TLR ligands (0.01 *μ*M−10 *μ*M). Tumor cells alone and together with PBL served as controls. 

**Figure 4:**
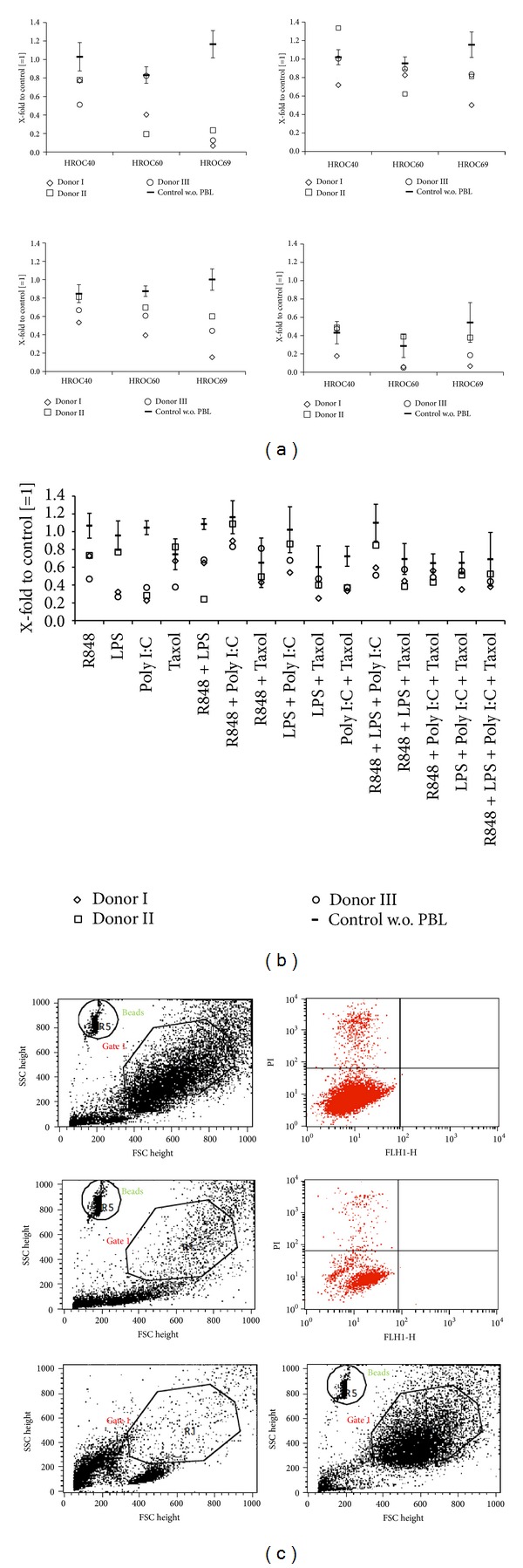
Coculture experiments. Tumor cells were cocultured with PBLs of three different healthy donors in the presence of (a) single TLR ligands (10 *μ*M) or in the presence of (b) TLR ligand combinations (0.01 *μ*M) for 72 h. Thereafter, numbers of viable tumor cells were quantified by flow cytometry using microsphere beads as calibrator. Tumor cells without TLR ligand were set as 1 and all other data were given as X-fold increase. (b) Results for single substance treatment (0.01 *μ*M) are additionally shown. ((a), (b)) For each approach, cells treated with the particular TLR ligand but without PBL are shown. (c) Representative dot plot illustrating the gating strategy for quantification of tumor cells. Shown are FACS data of HROC69 cells treated with PBL alone (control, upper panel) and treated with PBL + R848 (10 *μ*M, middle panel) for 72 h. Dead cells present in the gated tumor cells (Gate1) were excluded by staining with propidium iodide (PI^+^ cells; upper left quadrant in the right upper and middle blots). To illustrate the reliability of the gating-based separation of tumor cells and lymphocytes after coculture, FACS data of PBL alone and HROC69 cells alone are shown in addition (lower panel).

After 48 and 72 h of incubation, numbers of surviving tumor cells were determined using flow cytometry. Two of the donor's PBL showed strong reactions towards the CRC cell lines even in the absence of TLRs which must be considered as alloreactivity. Consequently, these were excluded from further analysis. Additionally, the results from the experiments without PBL addition are given to simplify comparability (Figure 4). 

Activation of immune cells by R848 resulted in strong cytotoxicity towards HROC69 (76%–93% killing versus control (tumor cells + PBL); Figure 4(a)). In this setting, a strong dose dependency could be observed with favorable effects in higher concentrations (data not shown). The CRC cell line HROC60 was moderately affected by R848 while HROC40 showed no susceptibility to immune-mediated antitumoral effects. The TLR ligand LPS exerted no effect on tumor cells in this coculture experiment. Poly I:C treatment led to reduced cell numbers when incubated with PBL and HROC69 (versus control: tumor cells + PBL). Interestingly, addition of PBL to tumor cells in the presence of the chemotherapeutic agent Taxol mediated strong oncopathic effects (Figure 4(a)). Numbers of surviving tumor cells fell to 5% compared to the control (tumor cells + PBL), and hence this coculture setting even enhanced the strong cytotoxic effects achieved by monotherapy (for comparison, please see Figure 1(d)).

Taken together, these data indicate elevated antitumoral effects by TLR ligands due to immune cell stimulation. However, tumor-cell-specific differences in vulnerability towards immune-mediated lysis were aparent. Taxol was the only substance leading to appreciable levels of cell number reduction for all cell lines.

Next, combinations of TLR ligands were added to tumor and immune cells. To identify potential synergistic effects, all possible TLR ligand combinations were considered (concentration of each TLR ligand: 0.01 *μ*M). Exemplary results are given in Figure 4(b). In this setting, most pronounced effects were obtained after Taxol/Poly I:C treatment, which was, however, comparable to Taxol monotherapy. Hence, TLR-combinations did not enhance immune-mediated oncolysis. By contrast, some combinations even tended to dampen antitumor responses (e.g., R848 + Poly I:C).

### 3.5. Impact of TLR Ligands on CRC Tumor Growth *In Vivo*

To finally prove the antitumoral effects of TLR ligands on CRC, an *in vivo* experiment was performed using the well-established CT26 tumor model. *In vitro*, this murine CRC cell line was sensitive towards Taxol but to no other TLR ligand used in this study (data not shown).

**Figure 5:**
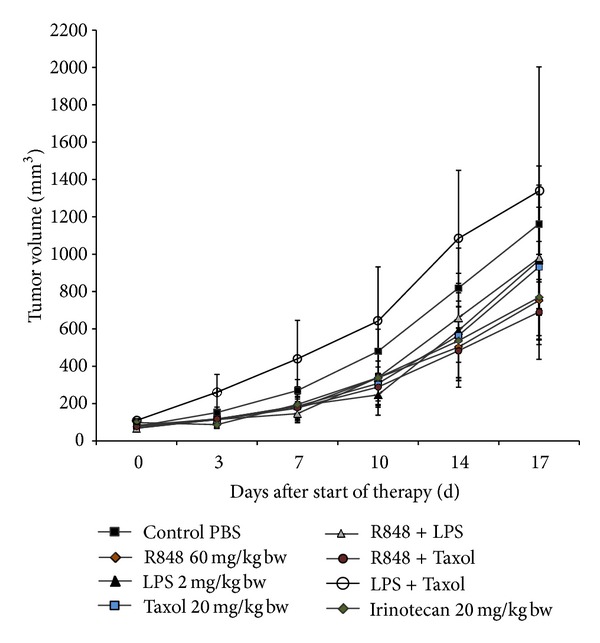
Tumor growth control *in vivo*. Growth kinetics of CT26 tumors in animals following injection of TLR ligands, their combinations, or Irinotecan. Therapy was performed by repetitive i.p. application of substances twice a week for a total of six times (*n* = 7). Control animals received equivalent volumes of PBS (*n* = 7). Values are given as mean ± SEM. None of the treatments reached statistical significance (*P* values d17 versus control: R848 0.232; Taxol 0.655; LPS 0.705; Irinotecan 0.340; R848 + Taxol 0.252; R848 + LPS 0.680; Taxol + LPS 0.789).

When tumors reached 50–100 mm^3^, experimental treatments were performed by biweekly i.p. applications of Taxol, R848, LPS, or TLR combinations (Figure 5). In order to better appraise TLR-mediated growth inhibition in this model, one group of animals was treated with the topoisomerase I inhibitor Irinotecan, a clinically approved drug that is standard to treat CRC patients. Control mice received equivalent volumes of the solvent alone (saline).

All treatment protocols, except two (Poly I:C and the combination of Taxol + LPS), mediated at least slight growth inhibition. R848 had strongest antitumoral potential within the TLR ligand monotherapy groups (compared to Taxol > LPS, versus control). Rather unexpectedly, Poly I:C exhibited a very strong tumor growth promoting activity and animals had to be redeemed by suffering at day 7 of therapy. The strongest antitumoral effects were obtained following Taxol and R848 therapy. This combination was even better in controlling tumor growth than Irinotecan. The combination of R848 + LPS slightly prevented tumor growth. However, no additive or synergistic effects could be obtained compared to single LPS or Taxol therapy.

### 3.6. Correlation of Immune Status with Course of Tumor Growth

**Figure 6:**
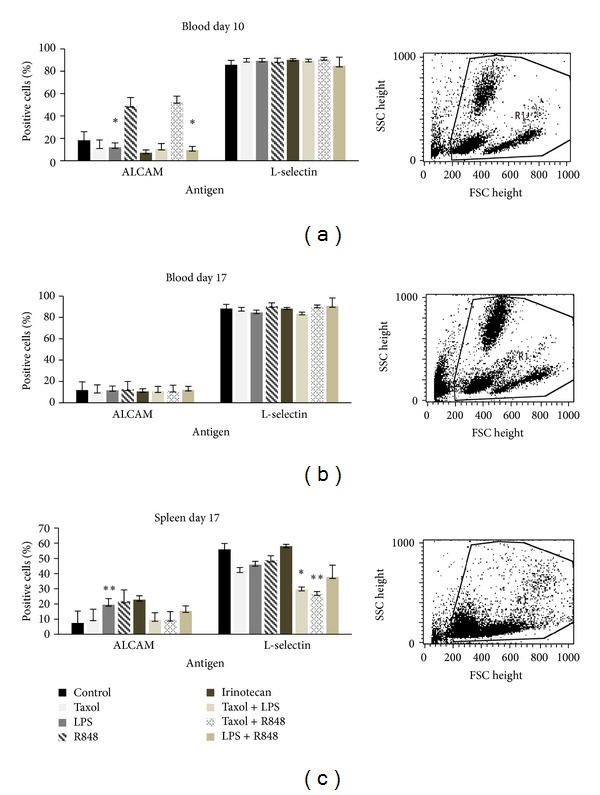
Analysis of leukocytes (a) during therapy and ((b), (c)) after necropsy from Balb/c mice. Blood samples of animals were taken on day 10 after start of therapy. At necropsy, blood samples and spleens were obtained and analyzed by flow cytometry. Given are the percentages of CD166 and CD62L positive cells. Control animals received equivalent volumes of PBS (*n* = 7). Values are given as mean + SD; *P* < 0.05 versus saline; *t*-test. Significant data for CD166 (ALCAM) were achieved in the blood at d10 in animals treated with Irinotecan (*P* = 0.032) and R848 + LPS (0.035) and additionally in the spleen at d17 in the LPS group (*P* = 0.004). For CD62L (L-Selectin), significance was reached in the spleen at d17 for R848 + Taxol (*P* = 0.002) and Taxol + LPS (0.038).

Finally, the involvement of the immune system in tumor growth control *in vivo* was examined. Blood samples from treated and control animals were analyzed on day 10 after start of therapy (Figure 6(a)) and upon therapy completion at day 17 (Figure 6(b)). Additionally, potential activation of immune cells in spleens of treated animals was studied (Figure 6(c)).

None of the treatment protocols mediated significant immunological changes, except for a massive increase in activated circulating CD166^+^ (ALCAM) immune cells accompanied by decreased levels of CD62L (L-selectin) cells. Especially significant elevations of CD166^+^ cells could be observed in the blood after 10d for treatment with Irinotecan (*P* = 0,032) and R848 + LPS (*P* = 0,035) as well as for LPS (*P* = 0,004) at day 17 in the spleen. Additionally, CD62L elevation achieved significance at day 17 in the spleen for the treatment groups R848 + Taxol (*P* = 0,002) and Taxol + LPS (*P* = 0,038). Both markers indicate T-cell activation. In case of L-selectin, proteolytic cleavage of cell surface molecules (=L-selectin shedding) or downregulation on the mRNA level possibly explains this observation best. Interestingly, this finding correlates with the antitumoral *in vivo* results, indicating involvement of these cell populations in tumor growth control.

However, we did not observe any other immune stimulatory effects at the given time points.

## 4. Discussion

In recent years, the old concept of fighting tumors with microbial agents has been rekindled by us and others [15–20]. This idea is to induce tumor regression both by direct oncolysis and indirectly through immune stimulation. To develop the approach further, we here explored the potential of defined TLR ligands as therapeutic agents. Therefore, we chose ligands for TLR3 (Poly I:C), TLR4 (LPS, Taxol), TLR7, and TLR8 (both R848). Those TLR ligands underwent extensive clinical investigations, clinical investigations. However, most studies focused on the immunostimulatory capacity of these molecules and their application as adjuvants along with tumor and virus vaccines [21–23]. Mechanistically, TLR ligands exert their antitumoral effect via activation of several cell types, including DCs and T cells [24, 25]. Due to their supposed direct antitumoral potential, TLR ligands are now tested as immunotherapeutic agents as well [26]. 

First, expression of relevant TLR receptors was analyzed on our freshly established, ultra-low-passage CRC tumor cell lines. Though expression pattern differed between cell lines, three out of the four receptors were detected (TLR3, 4, and 7). As expected, expression levels were comparably low (i.e., versus immune cells). By using cell lines in low passage (<40), most characteristics of primary tumor cells are retained. In those, varying TLR patterns have been found not only among normal/neoplastic cells (e.g., upregulation of TLR3/4 in tumors), but also within a single tumor [27]. However, this implies an important albeit unclarified role of TLRs in CRC biology. Therein, TLR expression is special, since the colon is continuously subjected to bacterial antigens and live bacteria. There is a fine line between physiological homeostasis of the commensal flora and stimulating tumor growth under conditions of chronic inflammation [28].

To shed light on the direct effect of TLR activation, primary CRC cells were here treated with the respective TLR ligands. Those were applied as single agents or in combinations to (i) mimic whole bacteria/viruses and (ii) to analyze if any synergisms in antitumoral action can be achieved. None of the employed substances mediated significant growth inhibition or tumor apoptosis/necrosis—neither as single agent nor in combination—some of them even tended to promote tumor growth (e.g., R848 + LPS; Figures 1 and 2). The sole exception was Taxol, a widely-used chemotherapeutic drug that additionally binds to TLR4. This finding is somehow interesting, since TLR4 expression often correlates with chemoresistance and metastasis [29]. Though we also observed interindividual differences, all cell lines responded to this drug (Figure 1). Antitumoral effects were slightly enhanced by adding another TLR ligand (Figure 2). However, combinations without Taxol did not mediate growth inhibition. This observation fits well with recent data from the literature. Emerging evidence suggests a dual role of TLR ligands, in which they simultaneously trigger both pro- and antitumoral effects depending on the applied molecule [7, 12, 13, 30]. In multiple myeloma cells, induction of autocrine interleukin-6/-18 production accounts for enhanced proliferation upon TLR activation [30, 31]. O'Leary and coworkers even described increased metastatic potential of colon cancer cells upon LPS stimulation via Nox1-mediated redox signaling [32]. By contrast, flagellin-induced TLR5 activation mediates tumor regression [33].

Despite their conflicting direct effects on tumor cells, TLR ligands are, to the most part, potent immune stimulators. In line with this well-established fact, we here observed boosted antitumoral effects in an *in vitro* coculture setting resembling aspects of a competent immune system (Figure 3). Effects were cell-line and substance specific—HROC40 cells generally tended to be more resistant than the other cell lines. Of note, R848 and Taxol proved most effective in stimulating immune-mediated tumor cell lysis, while LPS and Poly I:C did not work in our hands. Their supposed immune-stimulating potential was presumably not strong enough to negotiate the tumor cells' natural immunosuppressive capacity [34].

The TLR7/8 activator R848 is clinically approved for immunotherapy of skin tumors [35]. In patients, R848 treatment induces inflammatory cytokine secretion by macrophages and myeloid DCs as well as IFN-*α* release by plasmacytoid DCs [36]. Additional mechanisms include activation of NK cells. Besides, Taxanes mediate immunostimulatory effects against neoplasms, supporting the idea of a TLR ligand tumor vaccine. Experiences gathered in clinical studies demonstrated that Taxol enhances NK- and lymphokine activated killer cell functions [37]. The observed oncolytic effects in the present study were most likely also due to activation of NK or NK-like cells, whose tumor attacking potential is widely accepted [38–40]. 

To test this theory, immunotherapy with TLR ligands was performed in a syngeneic tumor model. Mice challenged with murine CT26 tumor cells received repetitive injections of TLR ligands. The route of application and intervals of treatment have been found to be crucial for an effective therapeutic schedule [36]. Topical application of TLR ligands is usually highly effective, whereas their systemic application met with limited success [41, 42]. However, for potential clinical application in CRC patients, systemic, repetitive injection is the method of choice. With this regimen, we observed at least partial growth retardation in our tumor model (Figure 4). Of note, monotherapy with R848 was as effective as Irinotecan, a first- and second-line standard therapeutic for advanced or recurrent CRCs [43]. The best combination used here was made of Taxol and R848, yielding >50% growth inhibition. Antitumoral effects were accompanied by massively increased levels of activated circulating CD166^+^ immune cells, that is, activated T cells and monocytes (Figure 5). This finding is consistent with the *in vitro* coculture results on human CRC and immune cells. Based on these *in vitro* findings, far better antitumoral results may be expected when testing this treatment approach in humanized mice, since TLRs are differently expressed in mice and humans [44]. This was also true for our human and mouse CRC cell lines. The exact mechanism of how Taxol and R848 act complementary and mutually reinforce antitumoral responses remains elusive. One may speculate that Taxol primarily inhibits direct tumor growth by interfering with the cell cycle. R848 on the other hand stimulates the immune system (primarily CD166^+^ cells). Both agents boost antitumoral immune responses that finally control tumor growth. 

A rather unexpected finding of the current study was the tumor-promoting activity of Poly I:C and the combination of Taxol + LPS. This was evident from the beginning of therapy. Tumors rapidly grew, became necrotic, and tended to ulcerate. In case of Taxol + LPS, this may best be explained by some kind of antagonism, in which both substances compete for the same TLR or intracellular signaling. Also, tumor or immune cells may respond with secretion of tumor-growth-promoting and immunosuppressive cytokines (e.g., IL10) [45]. These mechanisms abrogate the antitumoral effects of the single substances and strengthen tumor development. 

Therefore, TLR tolerance, characterized by a state of immune unresponsiveness, can be waived [36]. Moreover, since this was in contrast to the *in vitro* results, we can only speculate that the reasons for fostering of *in vivo* tumor growth by combinatorial treatment with these agents lie in the specific inter- and intracellular environment or may partly be attributable to the differences between human and mouse TLRs. 

Lastly, though TLR ligands are critical for first-line tumor therapy, there are many arguments in favor for their immunotherapeutic application: (i) single substances or combinations are ideal immune stimulators: both antigen-presenting (especially DCs) and effector cells (CD8^+^ T and NK cells) are functionally activated; (ii) conjugation to antigenic peptides is technically easy to perform; (iii) antibody-mediated cellular cytotoxicity is enhanced by increasing Fc-*γ* receptor expression; and thus treatment with monoclonal antibodies might be improved; and (iv) given their synthetic nature, they can be produced under GMP conditions and as a matter of fact, most ligands are already clinically approved.

## 5. Conclusion

Data presented herein prove the therapeutic potential of TLR agonists mediating both tumor inhibition and activation of immune effectors. Thus, they are very promising candidates for optimization of immune-based therapies, including applications as single agents or in combinations for active unspecific therapies, adjuvant standard regimens or in addition to cell-based immunotherapies. Our data also concern the Janus face character of TLR agonists and subsequent studies will further elucidate the exact balance between pro- and antitumoral activities of TLR agonists as single agents but especially of combinations.

## Figures and Tables

**Table 1:** TLR expression on CRC cell lines and immune cells.

	TLR3	TLR4	TLR7	TLR8
HROC40	++	++	+	−
HROC60	++	++	+	−
HROC69	−	++	+	−
DC	+++	+++	+++	+++
CT26	+++	+	+	n.d.
Macrophages	+	+++	+++	n.d.

ΔCT values were compared between cell lines and different target genes (+++ strong expression, ++ moderate expression, + low expression, − no expression, and n.d. not done).

## Abbreviations

PBL: Peripheral blood lymphocytes
TLR: Toll-like receptor
CRC: Colorectal carcinoma
DC: Dendritic cell.
